# Application of an Optimized Direct Lysis Method for Viral RNA Extraction Linking Contaminated Dates to Infection With Hepatitis A Virus

**DOI:** 10.3389/fmicb.2020.516445

**Published:** 2020-09-15

**Authors:** Sheikh Md Rajiuddin, Sofie Elisabeth Midgley, Tenna Jensen, Luise Müller, Anna Charlotte Schultz

**Affiliations:** ^1^Division of Microbiology and Production, National Food Institute, Technical University of Denmark, Kongens Lyngby, Denmark; ^2^Department of Virus and Specialist Microbiological Diagnostics, Statens Serum Institut, Copenhagen, Denmark; ^3^Division for Food and Feed Safety, Danish Veterinary and Food Administration, Glostrup, Denmark; ^4^Department of Infectious Disease Epidemiology and Prevention, Statens Serum Institut, Copenhagen, Denmark

**Keywords:** PCR, sequencing, outbreak, jaundice, foodborne, HAV

## Abstract

Consumption of dates has not been considered a common risk of hepatitis A virus (HAV) infection. In January 2018, an outbreak of hepatitis was identified with cases resident in all regions of Denmark. All the detected strains belonged to HAV genotype 3A. Epidemiological investigations through patients’ interviews, case-control and trace-back studies pointed toward different batches of dates from a single producer as the vehicle of infection. Boxes of dates from suspected batches were collected from homes of patients and healthy families and analyzed using a recently reported optimized direct lysis method, consisting of simultaneous viral RNA elution and extraction from dates followed by purification of the nucleic acids. Extracts were analyzed for HAV and norovirus (NoV) RNA using RT-qPCR, while detected HAV were genotyped by Sanger sequencing. Among 20 nucleic acid extracts representing eight batches of dates, RNA of HAV (9.3 × 10^2^ genome copies/g) and NoV genogroup (G)II (trace amounts) were detected in one batch, while NoV GII RNA (trace amounts) was detected in another. Average extraction efficiency of spiked process control murine norovirus was 20 ± 13% and the inhibitions of RT-qPCR detection of NoV GI, NoV GII, and HAV were 31 ± 34, 9 ± 9, and 3 ± 7%, respectively. The HAV genome detected in the dates matched by sequence 100% to the HAV genotype 3A detected in stool samples from cases implicated in the outbreak. This confirmed, to our knowledge, for the first time a sequence link between HAV infection and consumption of contaminated dates, suggesting dates to be an important vehicle of HAV transmission.

## Introduction

Hepatitis A virus (HAV) is one of the causative agents of viral jaundice and transmits primarily via the fecal-oral route through person-to-person contact or through contaminated food and water (Hollinger and Martin, 2013). Due to the ability to resist drying, freezing, food preservatives and acidification, HAV is highly persistent in the environment and has frequently caused foodborne disease outbreaks among susceptible populations worldwide (World Health Organization [WHO], 2017).

In recent years, consumption of HAV contaminated fruit imported from endemic areas has been identified as the source of several large and sometimes multistate outbreaks of HAV in Europe (EFSA, 2014; Severi et al., 2015). For example, HAV contaminated frozen berries caused 1,589 cases including two deaths in 13 EU/EEA countries during 2013–2014 (Severi et al., 2015), 71 cases in four Nordic countries including Denmark during 2012–2013 (Lassen et al., 2013) and 14 cases in the Netherlands in 2017 (Mollers et al., 2018). Consumption of semidried tomatoes has also been linked to outbreaks of HAV in Australia in 2009, and in the Netherlands and France in 2010 (Petrignani et al., 2010; Gallot et al., 2011; Donnan et al., 2012).

Attempts to detect and characterize HAV in suspected food products, identified by case control studies, to conduct a genetic comparison between HAV strains identified in clinical and food samples have often been unsuccessful, although it is essential to verify the origin of the foodborne disease (Petrignani et al., 2010; Gallot et al., 2011; Collier et al., 2014). One reason for this can be the un-availability of relevant food samples for laboratory analyses due to long lag time (2–6 weeks) between infection and onset of symptoms (EFSA, 2014). Other reasons may include uneven distribution of virus in suspected food, and low titer or purity of extracted viral RNA for un-inhibited HAV detection by reverse transcription (RT)-qPCR and characterization by sequencing (Schrader et al., 2012; EFSA, 2014).

An ISO standard for RT-qPCR detection of NoV and HAV in food matrices, including berries, ISO 15216-1, 2017 was recently validated (ISO, 2017). The method contains a protocol for virus and nucleic acid extraction based on elution of virus particles from food and capsid disruption using a chaotropic agent followed by nucleic acid adsorption to silica particles. The protocol includes quality control to identify false-negatives and prevent underestimation of viral load by evaluating the efficiency of viral RNA extraction using a virus process control, and amplification efficiency of target viral RNA by addition of an RNA control sequence to the RT-qPCR reaction. Altogether, the method is laborious and complicated, and needs to be simplified and optimized to increase viral recovery and purity of extracts (Bosch et al., 2018). In addition, the ISO standard is only validated for detection of NoV and HAV in the matrices described (soft fruit, leafy greens, stem and bulb vegetables, bottled water, mussels and oysters), excluding other types of potential vehicles of virus transmission, which limits the scope of the standard.

Perrin et al. (2015) proposed a direct viral lysis method from soft-fruit by immersion in lysis buffer, which enabled efficient detection of NoV surrogates on spiked raspberries, although it was less efficient in analyzing strawberries for the detection of spiked NoV (Bartsch et al., 2016). A slightly modified version in which inhibition could be overcome by filtering the RNA extract before molecular detection, confirmed the applicability of the method principle on samples of different berry types spiked with model viruses (Sun et al., 2019). However, the applicability of the direct lysis methods to detect foodborne viruses in naturally contaminated food samples remained to be demonstrated (Bosch et al., 2018).

To address the analytical challenge, we recently reported a further step toward improvement of the direct lysis method to allow for higher recovery of the process controls (mengovirus and murine norovirus, MNV) and less inhibited RT-PCR detection of the target viruses (NoV and HAV) in extracts from 13 different types of foods; fruits (including dates), vegetables, a compound food (seaweed salad) and seeds/nuts. The performance of the optimized direct lysis method was compared against a modified version of the ISO 15216-1 standard, and demonstrated successful detection of NoV in naturally contaminated strawberries and seaweed salad (Rajiuddin et al., 2020). However, the method’s broadness in detecting natural contamination remains to be shown for other viral pathogens and food matrices (Rajiuddin et al., 2020).

In January 2018, a hepatitis A outbreak was identified by RT-qPCR detection of HAV RNA in stool samples from 19 of 31 cases showing clinical symptoms of hepatitis infection from all regions of Denmark. Sanger sequencing and phylogenetic analysis grouped all the detected strains to HAV genotype 3A. Epidemiological investigations through patient interviews as well as case-control and trace-back investigations pointed toward different batches of dates from a single producer as the common route of exposure and thus the suspected vehicle of infection (Müller et al., 2018).

This study aimed to analyze samples of dates suspected to be implicated in the identified hepatitis A outbreak using a modified direct lysis method for the extraction of HAV RNA. In case of successful HAV detection, the aim was further to quantify the contamination level and to sequence the strain(s) detected in the date sample(s) in order to establish a match to the strains detected in the patient samples. Finally, the study aimed to explore the presence of NoV in the date samples, to evaluate a possible fecal contamination, in the case of unsuccessful HAV detection.

## Materials and Methods

### Date Samples

Ten boxes (box 1–10) of eight different batches (batch 1–8) of dates from the same brand and producer, were collected from the homes of four patients and from the homes of five families that contacted the Danish Veterinary and Food Administration (DVFA) offering the remaining dates for analyses in the wake of the preventive recall of the product. The dates were sent to the National Food Institute, Technical University of Denmark (DTU), for analysis for the presence of HAV and NoV.

### Elution, Extraction and Purification of Viral RNA From Date Samples

Viral RNA was extracted from 2 × 25–35 g (1–2 pieces) samples of each box of dates. To quality assess the extraction performance and control for false-negatives, the dates were spiked with MNV (10^4^ pfu or 10^5^ RT-qPCR units), propagated and titrated by plaque assay or end-point RT-qPCR, respectively (Rawsthorne et al., 2009; Hwang et al., 2014), and left to dry at room temperature for 30 min on a sterile bench.

Initially, box 1–2 were analyzed according to a viral RNA extraction method (Rajiuddin et al., 2020) structurally similar to the ISO 15216-1 (ISO, 2017) standard.

Subsequently, dates from box 1–10 were analyzed using a modified direct lysis method (Rajiuddin et al., 2020). Briefly, viral elution and simultaneous degradation of pectin and other potential RT-qPCR inhibitory substances, were conducted by inverting each sample 2–3 times in lysis buffer (10 ml) (NucliSENS^®^, bioMerieux, 280134) with the addition of pectinase (1 ml ∼3800 U) (Sigma, p2611 > 3,800 U/ml) and Plant RNA Isolation aid (400 μl) (Invitrogen, AM9690) followed by 10 min shaking at 200 rpm at room temperature. After incubation, the liquid buffer was separated from the solid date tissue by pipetting and centrifuged at 10,000 × *g* for 10 min at room temperature to pellet debris and small fruit particles. The supernatant (∼10.5–11.5 ml) was collected by pipetting and immediately used for nucleic acid extraction. Nucleic acids from all viral suspensions obtained by either method described above were extracted from the supernatant using the NucliSENS^®^ miniMAG^®^ system (BioMérieux, Herlev, Denmark) according to the manufactures instruction, except for using 140 μl of magnetic silica particles (BioMérieux, 280133) to catch released nucleic acid, and 100 μl of washing buffer three for the elution of purified RNA.

For the extracts obtained by the direct lysis method, a further purification of the nucleic acids was conducted using an OneStep PCR Inhibitor Removal Kit (Zymo Research, D6030) according to manufacturer’s instruction. Briefly, the *Prep-Solution* (600 μl) was added into a Zymo-Spin^TM^ III-HRC column placed in a *collection tube* beforehand and centrifuged at 8000 × *g* for 3 min. The spin column was transferred to a 1.5 ml microcentrifuge tube and 100 μl of previously extracted nucleic acid extracts from the date samples was transferred to the prepared column and centrifuged at 8000 × *g* for 1 min. The filtered RNA sample was preserved at 4°C for maximum 24 h or at −80°C for longer before being used for RT-qPCR analysis.

### Detection and Quantification of Viral RNA

Each extract was analyzed in duplicate for quantitative presence of HAV, NoV GI, and GII, as well as of MNV by RT-qPCR in a 96-well plate (MicroAmp Fast Optical 96-Well Reaction Plate 0.1 ml, Applier Biosystems, 4306311) on an ABI StepOnePlus Real-Time PCR machine (Applied Biosystems, Naerum, Denmark).

RT-qPCR reactions were carried out in a total of 25.0 μl mixture containing 5.0 μl of nucleic acid extract and 20.0 μl of master mix, including 1 × RNA Ultrasense One-Step Quantitative RT-PCR System (Thermo Fisher Scientific, Hvidovre, Denmark), and primers and TaqMan probes (DNA Technology AS, Risskov, Denmark) for NoV GI and NoV GII (Le Guyader et al., 2009), HAV (Costafreda et al., 2006) or MNV (Rawsthorne et al., 2009), and reaction conditions described by Le Guyader et al. (2009).

The theoretical limit of detection (tLOD) of the method was calculated to 20 genome copy (GC)/25 g date or 0.8 GC/g date. This was done by taking into account the volume changes and assuming that at least one GC of viral target (HAV or NoV) must be present in a positive RT-qPCR reaction containing 5 μl tested of total 100 μl extract obtained by processing a sample of 25–35 g date.

Quantification was performed using standard curves generated from 10-fold dilution series of artificially constructed dsDNA of HAV HM-175, NoV GI.1b, and NoV GII.4 (kindly provided by Dr. Lowther, Cefas, United Kingdom), produced and applied according to ISO 15216-1 (ISO, 2017), and extracted RNA of MNV.

The extraction efficiencies of the date samples were calculated using the formula 100e^–0.6978ΔCt^ as previously described by Le Guyader et al. (2009), where ΔCt is the difference in Ct values of the MNV RNA recovered from spiked samples and from nuclease free water.

To assess the purity of the extracted nucleic acids, inhibition controls for the detection of target virus were performed on all extracts according to ISO 15216-1 (ISO, 2017), by adding 10^4^ genome copies (GC) of NoV GI, NoV GII, and HAV transcripts (curtesy of Dr. Lowther CEFAS, United Kingdom) to separate wells containing 5.0 μl-volumes of each nucleic acid extract and to nuclease-free water.

The percentage of RT-qPCR inhibition was calculated as quotient of the GC amounts recovered of spiked RNA transcripts from the extracts (GC extract) and nuclease-free water (GC control) using the following formula: Inhibition (%) of target sequence = [1−(GC control/GC extract)] × 100. In cases of calculated negative inhibition of target sequence, lower inhibition in sample extract than in control, the inhibition was indicated as 0.0%.

### Characterization of Detected HAV Sequences

HAV genotyping by Sanger sequencing was carried out as described previously (Lassen et al., 2013). Sequence assembly and genotyping was performed using BioNumerics v7.6 (Applied Maths) and phylogenetic analysis was carried out using MEGA 6.

## Results

### Detection and Quantification of Viral RNA in Date Samples

Twenty nucleic acid extracts were obtained using the direct lysis method. HAV (9.3 × 10^2^ ± 6.5 × 10^2^ GC/g) and NoV GII (estimated 8.1 ± 7.7 GC/g) were detected in both extracts of box 10, originating from batch 4, while NoV GII RNA (estimated 2.4 ± 0.5 GC/g) was detected in one of two extracts of box 6, originating from batch 1 (Table 1). Neither HAV nor NoV GII was detected in dates from the remaining eight boxes extracted with the direct lysis method (box 1–5 and 7–9) and NoV GI could not be detected in any of the samples.

**TABLE 1:** Detection of target viral RNA, extraction efficiencies and RT-qPCR inhibition in extracts from dates attained using the optimized direct lysis method and the modified ISO 15216-1 standard (ISO, 2017).

Box^1^ ID	Batch ID	Best before date	Optimized direct lysis method^2^								Modified ISO 15216-1 method^2^					
			Weight of sample tested (g)	Extraction efficiency of MNV (%)		RT-qPCR inhibition^3^ (%)			Detection of viral RNA^4^		Weight of sample tested (g)	Extraction efficiency of MNV (%)		RT-qPCR inhibition (%)		
				Undiluted	10x diluted	NoV GI	NoV GII	HAV	NoV GII	HAV		Undiluted	10x diluted	NoV GI	NoV GII	HAV
1	1	30.09.2018	25.4 ± 1.9	14.1 ± 4.9	32.0 ± 8.5	0.0 ± 0.0	0.0 ± 0.0	0.0 ± 0.0	−−	−−	25.1 ± 0.1	0.5 ± 0.3	0.6 ± 0.6	0.0 ± 0.0	0.0 ± 0.0	0.0 ± 0.0
2	2	30.03.2018	25.1 ± 7.0	26.2 ± 27.7	34.0 ± 37.9	0.0 ± 0.0	0.0 ± 0.0	0.0 ± 0.0	−−	−−	25.3 ± 0.2	0.9 ± 0.3	1.0 ± 0.3	0.0 ± 0.0	0.0 ± 0.0	0.0 ± 0.0
3	3	10.06.2018	25.5 ± 0.6	13.0 ± 4.0	17.4 ± 5.4	40.5 ± 33.0	5.2 ± 3.5	0.0 ± 0.0	−−	−−						
4	4	30.06.2018	21.9 ± 0.6	17.1 ± 3.2	35.5 ± 16.5	43.4 ± 45.5	11.0 ± 15.1	1.4 ± 2.0	−−	−−						
5	5	30.06.2018	23.7 ± 0.2	34.5 ± 8.3	55.9 ± 18.1	26.7 ± 11.0	5.6 ± 7.9	8.2 ± 9.5	−−	−−						
6	1	30.09.2018	21.3 ± 0.6	37.2 ± 9.6	39.2 ± 7.6	0.0 ± 0.0	3.6 ± 5.0	5.9 ± 8.3	+ −	−−						
7	6	30.06.2018	25.2 ± 0.5	9.7 ± 3.0	42.7 ± 12.1	79.7 ± 14.2	21.5 ± 3.1	15.0 ± 21.3	−−	−−						
8	7	10.05.2018	33.0 ± 0.1	13.8 ± 9.6	40.8 ± 13.7	73.8 ± 18.6	16.3 ± 6.9	1.8 ± 2.5	−−	−−						
9	8	31.12.2017	24.2 ± 3.4	22.7 ± 2.3	27.2 ± 6.6	66.2 ± 16.8	15.4 ± 5.3	1.2 ± 1.7	−−	−−						
10	4	30.09.2018	33.1 ± 3.6	10.8 ± 6.5	7.7 ± 5.3	3.0 ± 5.2	8.4 ± 11.9	0.0 ± 0.0	++	++						
Mean values				19.9 ± 13.3	33.3 ± 19.2	30.6 ± 34.2	8.7 ± 8.9	3.1 ± 7.2				0.7 ± 0.4	0.8 ± 0.6	0.0 ± 0.0	0.0 ± 0.0	0.0 ± 0.0

*Data are presented as the mean ± standard deviations (Mean ± SD). ^1^All boxes of dates had been opened at the place of consumers, except for box 5. Original content of dates was 600 g’s for box 3, 5, and 7 and 400 g’s for the remaining boxes. ^2^Two extractions were performed from each box of dates, and the detection of target viruses as well as the extraction efficiency and RT-qPCR inhibition were each determined by separate two RT-qPCR reactions per extraction. ^3^In cases of calculated negative inhibition of target sequence, lower inhibition in sample extract than in control, the inhibition was indicated as 0.0%”. ^4^Each symbol represents positive (+) or negative (−) detection of viral RNA in one of two extract. NoV GI was not detected in any extractions using the optimized direct lysis method, neither was HAV, NoV GI, or NoV GII using the modified ISO 15216-1 method.*

The mean RNA extraction efficiency of spiked MNV was 20 ± 13% (range 10–37%) and the RT-qPCR inhibition of target sequences, NoV GI, NoV GII, and HAV, were 31 ± 34% (range 0–80), 9 ± 9% (range 0–21), and 3 ± 7% (range 0–15), respectively (Table 1). Except for one of two extracts from each box 4 and 7–9 displaying 75–90% NoV GI RT-qPCR inhibition, all extraction efficiencies and percentages of inhibition in undiluted extracts obtained using the direct lysis method complied with the quality criteria of extraction efficiency (≥1%) and RT-qPCR inhibition (≤75%) described in the ISO 15216-1 (ISO, 2017), see Table 1.

Neither HAV nor NoV could be detected in four nucleic acid extracts processed from box 1 and 2 using the modified ISO 15216-1 method, resulting in mean 0.7 ± 0.4% MNV RNA extraction efficiency and 0 ± 0% RT-qPCR inhibitions for all target viruses (NoV GI, NoV GII, and HAV).

### Sequence Analysis

A sequence of 1228 nucleotides covering the entire VP1 gene was obtained for strain DTU69S1 (deposited in GenBank, accession number MT222962) detected in the dates. Initial BLAST analysis against the SSI internal HAV database, and the NCBI GenBank nucleotide database, revealed it to be of genotype 3A, most closely related to outbreak strains and strains originating in Afghanistan and Pakistan. Phylogenetic analysis of 19 Danish outbreak strains and one strain from a patient from another country, and DTU69S1 showed a 100% nucleotide match between DTU69S1 and both the Danish case from whom the HAV positive date box was collected and another patient known to have consumed dates from a different batch (Müller et al., 2018).

## Discussion

Despite frequent outbreaks of hepatitis A suspected to be following consumption of contaminated foods, it has rarely been confirmed by detection of HAV in the implicated food (Petrignani et al., 2010; Gallot et al., 2011; Collier et al., 2014). Food products incriminated in such outbreaks are often imported from HAV endemic areas to European countries with susceptible populations at high risk of developing severe disease symptoms following consumption (World Health Organization [WHO], 2010; Lassen et al., 2013).

For the first time a HAV strain from samples of a batch of dates implicated in a foodborne HAV outbreak could be detected. The strain was quantified, characterized and genetically linked with a 100% match to the HAV sequence isolated from stool samples of one of the Danish cases included in the outbreak and from one other patient. The detection of HAV in nucleic acid extracts from date samples processed using the optimized and simple direct lysis method demonstrates the method’s applicability for detection of HAV in naturally contaminated dates.

However, since extractions of the positive dates were not conducted using the modified ISO 15216-1 method (ISO, 2017), we cannot exclude that this could also have resulted in a similar outcome. This widely used EU standard method for detection of viruses on soft fruit can be inefficient in recovering RT-qPCR detectable viral RNA from different types of soft fruits (Bartsch et al., 2016; Rajiuddin et al., 2020) including dates (Boxman et al., 2012). The reason is that food matrices may challenge efficient viral elution from the matrix surfaces and contain organic and inorganic substances that interfere with RT-qPCR detection of target sequences (Schrader et al., 2012; Perrin et al., 2015). Although with few samples, this study indicated a poor extraction efficiency (average <1%) of viral RNA from dates using the modified ISO 15216-1 method (ISO, 2017) compared to the direct lysis method (average 20%), without compromising on the degree of inhibition (average 0%) obtained for the four samples analyzed using both methods.

Previous studies using alternative variations of a direct lysis methods for extraction of spiked NoV (Bartsch et al., 2016) or model viruses have reported challenges with RT-qPCR inhibition, which could be overcome by dilution or filtration of extracts before RT-qPCR detection (Sun et al., 2019). In our study an inhibition >75% was observed for only NoV GI RT-qPCR detection in four sub-extractions from the dates, which thus failed the quality criteria described in the ISO 15216-1 (ISO, 2017), although extracts from the remaining subsamples and a 10-fold dilution of the inhibited subsamples did comply with this criteria. It is likely that the use of digital PCR (Coudray-Meunier et al., 2015) as genome detection and quantification method would overcome potential challenge of inhibition in sample extracts. However, this was not explored in this study.

In a Dutch surveillance study of dates including 185 samples, two batches of dates were tested positive for HAV and genotyped to 1A using a simplified method containing elution and RNA extraction compared to the ISO 15216-1 method (Boxman et al., 2012). However, despite suspicion of HAV illness among date consumers in the population, it was not possible to establish a link between the batches of dates tested and the infected patients.

Due to the estimated low contamination level in foods and the low efficiency of common methods to recover viral RNA, the characterization by sequencing of detected viral RNA in food materials may often be more difficult than in human stool or blood samples (EFSA, 2014). Previously, we reported that the optimized direct lysis method applied in this study was significantly more efficient in recovering RNA of spiked model viruses on a selection of types of fruits compared to the ISO standard (Rajiuddin et al., 2020). In this study, the optimized direct lysis method proved fit for purpose to extract HAV RNA that allowed RT-qPCR detection and quantification as well as sequence characterization from samples of naturally contaminated dates suspected to have caused a disease outbreak. The relatively high viral recovery and purity of extract, likely attributed to the fewer steps and the use of the OneStep PCR Inhibitor Removal Kit (McKee et al., 2015; Fraisse et al., 2017), may have complimented the successful sequencing in the optimized direct lysis method.

Approximately 10^3^ GC/g of HAV and trace amounts of NoV GII (<10 GC/gRNA were detected in the positive date samples. Although detection of viral RNA do not necessarily reflect detection of infectious viral particles (de Roda Husman et al., 2009), the related illness amongst consumers of dates indicates that at least some of the detected HAV genomes originated from intact virions.

In HAV endemic countries, the routes of contamination and spread of HAV and NoV may be common, with sewage-polluted water as an example (FAO/WHO, 2008). Since HAV detection in foods implicated in disease outbreaks is often unsuccessful (Dentinger et al., 2001; Petrignani et al., 2010; Carvalho et al., 2012; Lassen et al., 2013), we analyzed the date samples for presence of NoV in addition to HAV, with the rationale that, should the detection of HAV be unsuccessful, detection of NoV may indicate fecal contamination. Indeed, NoV GII RNA was detected in dates from boxes with and without simultaneous detection of HAV and RNA. This suggests that if HAV detection fails during investigation of HAV suspected contaminated food samples, NoV may be used as indicator for human fecal contamination. With the lack of reported gastroenteritis with dates as the suspected vehicle, the detection of NoV RNA might represent degraded viruses or levels below the number that cause symptoms, which has been estimated to 18–1000 virus particles (Teunis et al., 2008). Due to the detected levels of NoV RNA being lower than required for successful sequencing and the lack of reported gastroenteritis suspected to be caused by dates, sequence characterization of the detected NoV RNA in the two boxes of dates was not attempted.

While dates from two boxes (box 10 and 6) representing two different batches (batch 4 and 1, respectively) tested positive for either both HAV and NoV RNA or only NoV RNA, the paired two boxes (box 1 and 4, respectively) originating from the same batches did not test positive for any virus. This indicates that viruses may be unevenly distributed in contaminated dates, as has been previously observed for viral contaminated raspberries (EFSA, 2014), semidried tomatoes (Donnan et al., 2012) and dates (Boxman et al., 2012). The uneven distribution of viral contaminated fruits underlines the importance of a sound sampling strategy, when conducting surveillance or outbreak studies of fruits suspected to be contaminated with viruses (Bosch et al., 2011).

The HAV genome detected in the dates matched by sequence 100% to the HAV genotype 3A detected in the stool sample collected from one case from whose household the specific date sample was obtained and to another non-related infected HAV patient who also had consumed dates from the same producer. This strongly indicates that these cases were infected after consumption of the contaminated dates, rather than the product was contaminated by the case wherefrom the dates were collected.

Although dates have not yet been described as a main risk factor for HAV outbreaks (EFSA, 2014), the present study suggest like the Dutch surveillance study (Boxman et al., 2012) that dates may prove to be an important vehicle of widespread, cross-border foodborne outbreaks of hepatitis.

In conclusion, use of an optimized direct lysis method for the extraction of viral RNA from dates suspected to be the source of a hepatitis A outbreak, allowed detection, quantification and characterization of a HAV strain matching a sequence detected among the outbreak cases. This establishes, to our knowledge, for the first time a direct sequence link between a hepatitis A infection and consumption of contaminated dates.

## Data Availability Statement

The raw data supporting the conclusions of this article will be made available by the authors, without undue reservation, to any qualified researcher.

## Author Contributions

SR: writing – original draft preparation. AS: funding acquisition and supervision. TJ, SM, LM, and AS: resources. All authors: conceptualization and methodology of the study, formal analysis and investigation, and writing, reviewing, and editing of subsequent versions.

## Conflict of Interest

The authors declare that the research was conducted in the absence of any commercial or financial relationships that could be construed as a potential conflict of interest.
